# The risk of atherosclerosis in patients with chronic kidney disease

**DOI:** 10.1007/s11255-013-0407-1

**Published:** 2013-03-13

**Authors:** Sylwia Olechnowicz-Tietz, Anna Gluba, Anna Paradowska, Maciej Banach, Jacek Rysz

**Affiliations:** 1 Department of Internal Medicine, Nicolas Copernicus Regional Specialist Hospital, Lodz, Poland; 2 Department of Nephrology, Hypertension and Family Medicine, WAM University Hospital in Lodz, Lodz, Poland; 3 Department of Hypertension, Medical University of Lodz, Lodz, Poland

**Keywords:** Atherosclerosis, Chronic kidney disease, Comorbidities

## Abstract

**Background:**

Chronic kidney disease (CKD) is becoming a serious health problem; the number of people with impaired renal function is rapidly rising, especially in industrialized countries. A major complication of CKD is cardiovascular disease. Accelerated atherosclerosis has been observed in early stages of renal dysfunction. The purpose of this study was to examine the relationship between the degree of renal insufficiency and both the prevalence and intensity of coronary artery disease (assessed on the basis of number of vessels with stenosis).

**Methods:**

446 individuals with both serum creatinine >120 μmol/l (men) or >96 μmol/l (women) and acute coronary syndrome were included in the study. All patients included in this analysis underwent urgent coronarography. Data concerning glomerular filtration rate (GFR), number of vessels with stenosis, hypertension, lipid disorders, creatinine concentration, C-reactive protein, glucose and lipid profile were analyzed.

**Results:**

This study confirmed that moderate to severe renal impairment is associated with accelerated atherosclerosis. Moreover, patients with GFR values below 60 ml/min/1.73 m^2^ are predisposed to accelerated, multivessel cardiovascular disease.

**Conclusions:**

GFR seems to be an independent risk factor for multivessel cardiovascular disease. Due to the fact that patients with renal dysfunction are at high risk of cardiovascular events, they should obtain optimal treatment resulting not only in kidney protection but also in the elimination of cardiovascular risk factors.

## Introduction

Chronic kidney disease (CKD) is becoming a serious health problem; the number of people with impaired renal function is rapidly rising, especially in industrialized countries [1]. Among the main reasons for this situation are the increase of life span and wide prevalence of concomitant diseases such as type 2 diabetes, hypertension and cardiovascular disease, all of which may increase the risk of chronic kidney disease occurrence.

A major complication of chronic kidney disease is cardiovascular disease [2]. In patients with chronic renal disease, accelerated atherosclerosis has been observed. It was demonstrated that atherosclerotic lesions developed in early stages of renal dysfunction. Moreover, intense thickening of the vascular wall of peripheral arteries resulting from enhanced calcification of arterial media was detected [2]. Increased risk of cardiovascular disease in patients with renal disease is the reason for their enhanced morbidity and mortality. The risk of death from cardiovascular disease (CAD) increases along with the decrease in renal function. A slight decline in glomerular filtration rate in the second stage of chronic renal disease results in two- to threefold higher risk of CAD; in dialyzed patients, this risk is increased from 10 to 100 times in comparison with the general population [3].

In patients with impaired renal function, classical cardiovascular risk factors are more common than in the general population. Hypertension (HA) is one of the main causes of chronic kidney disease and is present in 70 % of CKD patients and in 80–90 % of dialyzed patients [4, 5]. Also, dyslipidemia is highly prevalent among patients with CKD [6, 7]. Lipid disorders appear at the early stages of renal disease and as CKD progresses, they become more intense. Numerous studies have revealed that in CKD dialyzed patients, the relation between cholesterol level and mortality is frequently reversed, meaning that low levels of TCH are associated with higher cardiovascular mortality [8].

However, recent studies have suggested that in patients with chronic kidney disease, the increased occurrence of atherosclerosis is also associated with novel risk factors such as chronic inflammatory state, calcium–phosphate metabolism disturbances, oxidative stress, malnutrition, anemia, fluid overload, fluctuation in systemic fluid volume, disturbances in coagulation system, accumulation of metabolic products and numerous undefined toxic agents [9, 10].

The purpose of this study was to examine the relationship between the degree of renal insufficiency and both the prevalence and intensity of coronary artery disease (assessed on the basis of number of vessels with stenosis).

## Subjects and methods

This retrospective population-based study included 446 individuals (173 women and 273 men) with negative history of diabetes mellitus (DM) and disorders of carbohydrate metabolism who were admitted to the Dept. of Interventional Cardiology, Cardiodiabetology and Cardiac Rehabilitation WAM University Hospital of Lodz. Patients with both serum creatinine >120 μmol/l (men) or >96 μmol/l (women) and acute coronary syndrome (ACS) were included in the study group. Patients with DM, neoplastic and systemic diseases, and people in shock and with hyperthyroidism or hypothyroidism were excluded from the study. Estimated glomerular filtration rate (eGFR) was calculated for all subjects using the MDRD equation [11]. Serum creatinine was measured before percutaneous coronary intervention (PCI) and in some patients also after the procedure. On the basis of the eGFR, patients were divided into subgroups according to the stage of chronic kidney disease [12]. Data concerning the former occurrence of HA, lipid disorders and their treatment as well as smoking status were included in the analysis. Hypertension was diagnosed during patients’ stay in hospital according to ESH/ESC Hypertension Guidelines (>140 mmHg systolic and/or 90 mmHg diastolic pressure) [13] or on the basis of previous diagnosis and implemented hypertensive treatment. Lipid disorders were also diagnosed during patients’ stay in hospital or previous diagnosis according to ESC guidelines [hypercholesterolemia: total cholesterol (TC) > 190 mg/dl (>5 mmol/l), low density lipoprotein (LDL-C) > 115 mg/dl (>3 mmol/l); hypertriglyceridemia: triglycerides (TG) > 150 mg/dl (>1.7 mmol/l); mixed hyperlipidemia: TC > 190 mg/dl (>5 mmol/l), LDL-C > 115 mg/dl (>5 mmol/l) and TG > 150 mg/dl (>1.7 mmol/l); low concentration of HDL < 40 mg/dl (<1 mmol/l) in men, <45 mg/dl (<1.2 mmol/l) in women] [14].

Ischemic changes were detected on the basis of electrocardiogram. Data concerning concentration of creatinine, urea, C-reactive protein (CRP), glucose and lipid profile were obtained from patients’ hospital records. Moreover, in hospital, every patient underwent blood pressure measurement and anthropometric examination (e.g., weight, height, circumference of the abdomen) in order to calculate body mass index (BMI). All patients included in this analysis underwent urgent coronarography. On the basis of coronarography results, patients were assigned to subgroups according to the number of vessels with atherosclerotic lesions. Narrowing of more than 50 % of the lumen of coronary arteries (LCA, LCX and RCA) and 30 % or more of the left main coronary artery were treated as significant. The narrowing of LCA, LCX or RCA was treated as one-vessel disease, left main coronary artery stenosis or narrowing of two vessels as double-vessel disease and narrowing of three vessels or left main coronary artery stenosis and one vessel as 3-vessel disease.

Logistic regression was used to analyze the relationship between dichotomous-dependent variables and both continuous and discrete independent variables. Variables which significantly altered univariate model adjustment were included in the multivariable analysis. Since the relationship between GFR values and the risk of accelerated atherosclerosis in coronary vessels (on the basis of coronarography) was nonlinear, the analysis of logistic regression was supplemented with ROC curve analysis. Such analysis resulted in calculation of the GFR value at which this variable reached optimal resolution for the prediction of increased risk of intense atherosclerotic changes in coronary vessels. The results of logistic regression analysis were confirmed by testing the area under the ROC curve with the hypothetical value of 0.5. STATISTICA and SPSS PC programs were used for calculations. A significance level *α* < 0.05 was used in all tests.

## Results

Patients in the study group were on average 62.34 ± 10.84 years old (66.42 ± 10.30 women; 59.75 ± 10.38 men) with mean BMI 27.71 ± 3.86 (27.75 ± 4.30 women, 27.69 ± 3.59 men). Patients’ baseline clinical and biochemical characteristics are presented in Table 1.

**Table 1:** Characteristics of patients

	Sex	Mean	SD	Mean ± SD
Age	Women	68.03	10.01	64.05 ± 10.73
	Men	61.16	10.31	
BMI (kg/m^2^)	Women	28.80	6.00	28.49 ± 4.86
	Men	28.30	3.96	
Diabetes (%)	Women	30.28	–	26.3 %
	Men	23.3	–	
Hypertension (%)	Women	73.24	–	66.9 %
	Men	62.31	–	
Lipid disorders	Women	72.89	–	69.7 %
	Men	64.43	–	
Smoking status	Women	15.14	–	22.1 %
	Men	27.18	–	
HbA1c ≥ 6.1 %	Women	48.24	–	43.8
	Men	40.51	–	
CRP ≥ 6	Women	26.06	–	24.9
	Men	24.10	–	
Atherosclerotic lesions:		No.	%	%
No changes		65	9.6	9.6
In 1 vessel		193	28.6	90.4 %
In 2 vessels		195	28.9	
In 3 vessels		221	32.8	
MDRD-GFR before PCI	GFR range	No.	%	%
	≥90	36	5.3	47.1
	60–89	281	41.7	
	30–59	317	47.0	52.9
	15–29	25	3.7	
	<15	15	2.2	
MDRD-GFR after PCI	GFR range	No.	%	%
	≥90	27	6.5	48.8
	60–89	175	42.3	
	30–59	187	45.2	51.2
	15–29	17	4.1	
	<15	8	1.9	

The analysis of the association between GFR (CKD stage) and other variables revealed that HA was mainly present in stage IV CKD patients (100.0 % of patients in this group) and in those with atherosclerotic lesions in 3 vessels (69.6 %). It occurred rarely in patients with stage II CKD (51.4 %) and in those with atherosclerotic changes in 1 vessel (54.8 %). Lipid disorders were most frequent in patients with stage IV CKD (88.9 %) and in those with atherosclerotic changes in 1 or 3 vessels (76.7 and 76.5 %, respectively). It was less frequent in stage V CKD (37.5 %) and in patients with no changes in vessels (56.8 %). Nicotinism was observed mostly in patients with GFR above 90 ml/min/1.73 m^2^ (60.0 %) and in those with changes in 1 vessel (34.9 %). BMI ≥30 kg/m^2^ was most common among patients with stage IV and V CKD (33.3 and 33.3 %, respectively) and in those without any plaques in vessels (32.4 %). CRP above the upper limit was observed mainly in patients with stage IV CKD (77.8 %) and in those with atherosclerotic plaque in three vessels (35.1 %). Results are summarized in Table 2.

**Table 2:** The association between CKD stage and other variables

	CKD stage									
	I		II		III		IV		V	
	*N*	%	*N*	%	*N*	%	*N*	%	*N*	%
Diabetes mellitus (DM)	3	8.8	42	15.2	111	36.0	14	60.9	7	46.7
Newly recognized DM	1	2.8	16	5.7	16	5.0	0	0.0	0	0.0
HbA1c ≥ 6.1	10	29.4	99	36.8	161	58.1	18	75.0	7	58.3
BMI ≥ 30	7	22.6	77	31.8	89	39.4	9	60.0	2	22.2
Lipid disorders	28	77.8	219	77.9	225	71.0	17	68.0	9	60.0
Hypertension	20	55.6	159	56.6	252	79.5	24	96.0	12	80.0
Exposure to cigarette smoke	18	50.0	92	32.7	35	11.0	3	12.0	1	6.7
CRP > 6	7	21.2	52	20.2	87	35.8	18	81.8	4	50.0

On the basis of collected data, three logistic regression models were created. First, data were analyzed using a univariate logistic regression model, and then, multivariable models were created. In univariate models, the association between the number of vessels with atherosclerotic plaque (dichotomous-dependent variable) and independent variables such as age, sex, BMI, circumference of the abdomen, hypertension, lipid disorders, nicotinism, CRP level, creatinine level and GFR (every stage separately) was analyzed. The dependent variable could have one of two values: lack of changes in vessel or changes in at least 1 vessel in the first model; changes in 1 vessel or changes in at least 2 vessels in the second model; and changes in at least 2 vessels or changes in 3 vessels in the third model. Statistically significant associations were observed between the occurrence of atherosclerotic plaque in at least 1 vessel and age (*p* = 0.008), lipid disorders (*p* = 0.012), exposure to cigarette smoke (*p* = 0.014), between atherosclerotic plaque in at least 2 vessels and age (*p* = 0.008), circumference (*p* = 0.37), HA (*p* = 0.035), CRP > norm (*p* = 0.027), creatinine level > norm (*p* = 0.024), as well as between the occurrence of atherosclerotic plaque in at least 3 vessels and age (*p* = 0.026), HA (*p* = 0.037), creatinine level > norm (*p* < 0.001), GFR (continuous variable) (*p* = 0.001) and GFR (≤29 ml/min/1.72 m²) (*p* = 0.009) (Table 3).

**Table 3:** Results of one-factorial model of logistic regression for the occurrence of changes in at least 1, 2 and 3 vessels

Independent variables	OR	−95 % CI	+95 % CI	*p*
*At least one vessel*				
Age	1.041	1.010	1.072	0.008
Lipid disorders	2.265	1.197	4.287	0.012
Exposure to smoke	3.327	1.280	8.649	0.014
*At least two vessels*				
Age	1.027	1.007	1.047	0.008
Hypertension	1.565	1.031	2.374	0.035
Lipid disorders	0.856	0.532	1.376	0.520
Creatinine > norm	1.633	1.067	2.499	0.024
*At least three vessels*				
Age	1.024	1.003	1.045	0.026
Hypertension	1.635	1.030	2.595	0.037
CRP > norm	1.648	0.991	2.742	0.054
Creatinine > norm	2.323	1.494	3.613	<0.001
GFR (continuous variable)	0.980	0.969	0.992	0.001
GFR ≤ 29	7.333	1.633	32.925	0.009

To indicate independent risk factors for atherosclerotic plaque occurrence in at least 1, 2, 3 vessels, multivariable logistic regression models were created. Only in the case of 1 vessel, statistically significant relationships were found (Table 4). The risk of changes in vessels increases by 4.4 % with age. Smoking increases the risk of lesions in vessels 4.5-fold.

**Table 4:** Results of multifunctional model of logistic regression

Independent variables	OR	−95 % CI	+95 % CI	*p*
*At least one vessel*				
Age	1.044	1.010	1.079	0.011
Exposure to smoke	4.442	1.643	12.014	0.003

The association between the occurrence of atherosclerotic plaques and GFR (calculated before and after PCI) was analyzed by ROC curves. Values of area under ROC curves and the results of significance tests are summarized in Table 5. A statistically significant result was obtained only in the case of 3 vessels. This result is in accordance with the results of logistic regression analysis. On the basis of the ROC curve, the GFR value at which the test reached optimal resolution for the prediction of increased risk of intense atherosclerotic changes in coronary vessels was calculated. Patients with GFR equal to 54 ml/min/1.73 m^2^ were at highest risk of developing 3-vessel disease.

**Table 5:** Results of analysis of ROC curves (changes in 3 vessels)

Tested variable	Field	SD	Asymptotic significance	95 % Asymptotic confidence interval	
GFR before PCI	0.630	0.038	0.001	0.554	0.705
GFR after PCI	0.589	0.041	0.025	0.509	0.669

Using the calculated GFR, another multivariable logistic regression model was created. In this analysis, the independent variable GFR had one of the following values: GFR < 54 or GFR ≥ 54. Results of this analysis are presented in Table 6.

**Table 6:** Results of multivariable logistic regression model (changes in 3 vessels)

Independent variable	OR	−95 % CI	+95 % CI	*p*
GFR < 54	1.971	1.266	3.067	0.003

This analysis demonstrated the existence of one independent risk factor for the development of atherosclerosis in 3 vessels: GFR < 54 increases nearly twofold the risk of 3-vessel atherosclerosis in comparison with risk of 2-vessel disease.

## Discussion

Numerous epidemiological studies have analyzed risk factors associated with atherosclerosis development. Common atherosclerotic risk factors include age, sex (male), lipid disorders, HA, diabetes, obesity, smoking, lack of physical activity and also genetic predisposition. In this study, atherosclerotic risk was mainly connected with lipid disorders (71.3 %), HA (59.6 %), nicotinism (27.8 %) and obesity (22.6 %). The results of this study are in agreement with the POLKARD SPOK [15] study conducted on Polish population.

Recently, some studies have revealed that even a slight renal impairment manifested by microalbuminuria and increase in GFR poses an essential, but underestimated, cardiovascular risk factor. It is difficult to decide whether kidney disease itself is connected with the increased cardiovascular risk, since it is often accompanied by numerous atherosclerosis risk factors. It was observed that among people with impaired renal function, the frequency of cardiovascular disease occurrence is much higher than in the general population [16–19].

According to studies, cardiovascular events are 3.5–100 times greater in dialysis-dependent CKD patients than the general population [18, 19]. Patients with CKD have also much greater mortality from myocardial infarctions (MIs) [20, 21]. Wright et al. [20] demonstrated that the mortality of patients receiving dialysis reaches 60 % in the first year after a first MI, while in-hospital mortality after an MI is 2 % in subjects with normal renal function, 6 % with mild CKD, 14 % with moderate CKD, 21 % with severe CKD and 30 % in dialysis patients [20]. Also a large prospective population-based cohort study demonstrated a graded and independent association between renal function and the risk of MI in an elderly population [22]. The authors observed the association between mild renal insufficiency and increased risk of cardiovascular disease, which, as they suggested, could be explained by the fact that renal insufficiency itself might initiate and accelerate CAD [22]. According to the authors, the impact of renal insufficiency on the development of CAD may be greater than commonly thought. Thus, the assessment and treatment of decreased renal function at an early stage may prove helpful in the prevention of CAD [22].

This study demonstrated that 44.4 % of patients who underwent coronarography due to ACS had GFR < 60 ml/min/1.73 m^2^, meaning that nearly half of those patients were in at least stage III of CKD. These results are in accordance with the study of Othake et al. [23], who observed advanced atherosclerosis in dialyzed patients without any symptoms of ACS. Over half of them (53 %) had significant stenosis in coronary arteries (≥50 %). Single-vessel disease was diagnosed in 62.5 %, double-vessel disease in 25 % and 3-vessel disease in 12.5 %. Also Gradus et al. [24] reported an association between renal disease and the number of atherosclerotic lesions.

In patients especially with end-stage CKD, the number of co-existing cardiovascular risk factors is high. Apart from HA, in these patients, enhanced inflammatory and oxidative stress, anemia and calcium–phosphate metabolism disturbances have been observed. Such disturbances may lead not only to the progression of kidney disease, but also to accelerated atherosclerosis, calcification of lesions and intima-media thickening [9, 25].

Also, this study demonstrated that renal impairment is associated with the occurrence of classical atherosclerotic risk factors and with progression of atherosclerosis. The analysis of the association between GFR fraction and other variables revealed that lipid disorders occurred most frequently in patients with stage IV CKD and in those with atherosclerotic changes in 1 or 3 vessels. HA was mainly present in stage IV CKD patients and those with atherosclerotic lesions in three vessels. CRP levels exceeding the upper limit were observed especially in patients with stage IV CKD and in those with atherosclerotic plaque in 3 vessels. These results may suggest that risk factors co-existing with CKD promote atherosclerosis. Regression analyses revealed the association between the occurrence of atherosclerotic plaque and age, HA, lipid disorders, smoking status, CRP and creatinine and decreased GFR. Also, Khalique et al. [26] observed high prevalence of classical CAD risk factors in patients with CKD. In their study, hyperlipidemia was detected in 77 % of patients with GFR < 60 ml/min/1.73 m^2^, HA in 74 % and DM in 43 % [26].

Although high cardiovascular morbidity and mortality are observed in CKD patients, there are only a few clinical studies concerning this problem. Among 86 studies regarding various methods of CAD treatment published between 1998 and 2005, as many as 80 % of them excluded patients with end-stage renal disease and 75 % excluded patients in earlier CKD stages [27].

In this study, the influence of GFR on the occurrence of atherosclerotic lesions was analyzed using ROC curves. Statistically significant results were obtained only in the case of 3 vessels. Lack of association between GFR value and the occurrence of changes in at least 1 as well as in at least 2 vessels is in accordance with the results of logistic regression analysis. ROC analysis demonstrated that patients having GFR equal to 54 ml/min/1.73 m^2^ were at the highest risk of developing 3-vessel disease. Another analysis demonstrated the existence of only 1 independent risk factor for the development of atherosclerosis in 3 vessels. GFR < 54 increased the risk of 3-vessel atherosclerosis nearly twofold in comparison to 2-vessel disease. These results confirm the thesis that decreased GFR is an independent risk factor for 3-vessel disease. The risk of 3-vessel atherosclerosis in patients in stage IV and V of CKD is 7.3-fold higher than in patients with stage I CKD. This study confirmed that moderate to severe impairment of kidney function (stages III, IV, V) is associated with accelerated atherosclerosis and that GFR < 60 ml/min/1.73 m^2^ predisposes to the development of advanced, multivessel cardiovascular disease.

The number of publications concerning the connection between GFR value and the number of vessels with atherosclerotic plaque is very low. One of these studies [26] demonstrated that in patients with stage III, IV and V CKD, 1-vessel disease was present in 87 % of patients and 3-vessel disease in 53 % of patients. In the group of patients with slightly decreased GFR or with normal renal function, 71 % had stenosis in 1 vessel and only 23 % suffered from 3-vessel disease [26].

The increase in the risk of cardiovascular incidents in patients with mild renal impairment was also observed in other studies. According to Vanholder et al. [28], GFR < 75 ml/min/1.73 m^2^ is associated with higher risk of CAD and its further decrease by every 10 ml/min/1.73 m^2^ results in an increased risk of cardiovascular end-point events by 20 %. Gibson et al. [29] found that in patients with ACS without ST elevation (NSTEMI ACS), a decrease in renal function is linked to a higher percentage of 3-vessel disease and that eGFR is independently associated with 30-day and 6-month mortality [29].

This study confirmed that moderate to severe renal impairment (stage III, IV, V CKD by K/DOQI) is associated with accelerated atherosclerosis, and it demonstrated that eGFR < 54 ml/min/1.73 m^2^ is the strongest independent predictor of the risk of ACS in renal patients with three coronary vessels involvement. Moreover, patients with GFR values below 60 ml/min/1.73 m^2^ are predestined to accelerated, multivessel cardiovascular disease. GFR seems to be an independent risk factor for multivessel cardiovascular disease. Due to the fact that patients with renal dysfunction are at high risk of cardiovascular events, they should obtain optimal treatment resulting not only in kidney protection but also in the elimination of cardiovascular risk factors.

## Study limitations

A limitation of the study is the fact that serum creatinine was measured after acute coronary disease and the estimated GFR on admission was susceptible to the initial hemodynamic state of the patient and so could not accurately reflect baseline renal function. However, according to a study of creatinine level at admission, it is one of the most important covariates in early prognostic stratification of patients with non-ST-elevation acute coronary syndrome [30]. In this study, creatinine level was not the only parameter for the assessment of renal function. Another limitation is that data concerning proteinuria were not available in this study. Moreover, coronary calcification was not assessed and Ca and P ions, calcium–phosphorus product (CaxP) and parathyroid hormone (PTH) were not examined in this study. Moreover, there was no follow-up; thus, the information concerning possible CV complications in patients is not available.

## References

[1] Schieppati A, Remuzzi G (2005). Chronic renal diseases as a public health problem: epidemiology, social, and economic implications. Kidney Int Suppl.

[2] Campean V, Neureiter D, Varga I (2005). Atherosclerosis and vascular calcification in chronic renal failure. Kidney Blood Press Res.

[3] Levey AS, Beto JA, Coronado BE (1998). Controlling the epidemic of cardiovascular disease in chronic renal disease: what do we know? What do we need to learn? Where do we go from here? National kidney foundation task force on cardiovascular disease. Am J Kidney Dis.

[4] De Santo NG, Cirillo M, Perna A (2001). The heart in uremia: role of hypertension, hypotension, and sleep apnea. Am J Kidney Dis.

[5] Banach M, Rysz J (2010). Current problems in hypertension and nephrology. Expert Opin Pharmacother.

[6] Barylski M, Małyszko J, Rysz J, Myśliwiec M, Banach M (2011). Lipids, blood pressure, kidney—what was new in 2011?. Arch Med Sci.

[7] Gluba A, Rysz J, Banach M (2010). Statins in patients with chronic kidney disease: why, who and when?. Expert Opin Pharmacother.

[8] Kasiske BL (1998). Hyperlipidemia in patients with chronic renal disease. Am J Kidney Dis.

[9] Sarnak MJ, Levey AS, Schoolwerth AC (2003). Kidney disease as a risk factor for development of cardiovascular disease. A statement from the American heart association councils on kidney in cardiovascular disease, high blood pressure research, clinical cardiology, and epidemiology and prevention. Hypertension.

[10] Malyszko J, Bachorzewska-Gajewska H, Malyszko H, Iaina-Levin N, Kobus G, Dobrzycki G (2011). Markers of kidney function in the elderly in relation to the new CKD-EPI formula for estimation of glomerular filtration rate. Arch Med Sci.

[11] Levey AS, Coresh J, Balk E (2003). National kidney foundation practice guidelines for chronic kidney disease: evaluation, classification, and stratification. Ann Intern Med.

[12] National Kidney Foundation (2002). K/DOQI clinical practice guidelines for chronic kidney disease: evaluation, classification, and stratification. Am J Kidney Dis.

[13] Guidelines 2007 ESH-ESC Practice Guidelines for the Management of Arterial Hypertension. ESH-ESC Task Force on the Management of Arterial Hypertension. http://www.swisshypertension.ch/docs/2007_hypertension_practice_guidelines.pdf 10.1097/HJH.0b013e3282f0580f 17762635

[14] Graham I, Atar D, Borch-Johnsen K i wsp. (2007) European Guidelines on cardiovascular disease prevention in clinical practice. Fourth Joint Task Force of the European Society of Cardiology and other Societies on Cardiovascular Disease Prevention in Clinical Practice. Eur J Cardiovasc Prev Rehab 14 (Suppl 2): S11–113 10.1097/01.hjr.0000277983.23934.c9 17726407

[15] Pietrasik A, Starczewska M, Główczyńska R (2006). Secondary prevention of myocardial infarction in primary health care in Poland—selected results of the POLKARD-SPOK. Kardiol Pol.

[16] Skóra B, Gluba A, Banach M, Rozentryt P, Poloński L (2012). Rysz J.

[17] Couser WG, Riella MC (2011). World kidney day 2011: protect your kidneys, save your heart. Arch Med Sci.

[18] Brunner FP, Selwood NH (1992). Profile of patients on RRT in Europe and death rates due to major causes of death groups. The EDTA registration committee. Kidney Int Suppl.

[19] Parekh RS, Carroll CE, Wolfe RA, Port FK (2002). Cardiovascular mortality in children and young adults with end-stage kidney disease. J Pediatr.

[20] Wright RS, Reeder GS, Herzog CA (2002). Acute myocardial infarction and renal dysfunction: a high-risk combination. Ann Intern Med.

[21] McCullough PA, Sandberg KR, Borzak S, Hudson MP, Garg M, Manley HJ (2002). Benefits of aspirin and beta-blockade after myocardial infarction in patients with chronic kidney disease. Am Heart J.

[22] Brugts JJ, Knetsch AM, Mattace-Raso FUS, Hofman A, Witteman JCM (2005). Renal function and risk of myocardial infarction in an elderly population. The Rotterdam Study. Arch Intern Med.

[23] Ohtake T, Kobayashi S, Moriya H (2005). High prevalence of occult coronary artery stenosis in patients with chronic kidney disease at the initiation of renal replacement therapy: an angiographic examination. J Am Soc Nephrol.

[24] Gradaus F, Ivens K, Peters AJ (2001). Angiographic progression of coronary artery disease in patients with end-stage renal disease. Nephrol Dial Transplant.

[25] Gluba A, Mikhailidis DP, Lip GY, Hannam S, Rysz J, Banach M. Metabolic syndrome and renal disease. Int J Cardiol. 2012 Feb 2 10.1016/j.ijcard.2012.01.013 22305775

[26] Khalique O, Aronow WS, Ahn C (2007). Relation of moderate or severe reduction in glomerular filtration rate to number of coronary arteries narrowed >50 % in patients undergoing coronary angiography for suspected coronary artery disease. Am J Cardiol.

[27] Charytan D, Kuntz RE (2006). The exclusion of patients with chronic kidney disease from clinical trials in coronary artery disease. Kidney Int.

[28] Vanholder R, Massy Z, Argiles A, Spasovski G, Verbeke F, Lameire N, European Uremic Toxin Work Group (2005). Chronic kidney disease as cause of cardiovascular morbidity and mortality. Nephrol Dial Transplant.

[29] Gibson CM, Dumaine RL, Gelfand EV (2004). Association of glomerular filtration rate on presentation with subsequent mortality in non-ST-segment elevation acute coronary syndrome; observations in 13,307 patients in five TIMI trials. Eur Heart J.

[30] Fácila L, Núñez J, Bodí V, Sanchís J, Bertomeu-González V, Consuegra L, Pellicer M, Ferrero A, Sanjuán R, Llácer A (2006). Prognostic value of serum creatinine in non-ST-elevation acute coronary syndrome. Rev Esp Cardiol.

